# Issues of Renal Replacement Therapy in Elders Living Low-Income African Countries

**DOI:** 10.5812/numonthly.7077

**Published:** 2012-09-24

**Authors:** Sidy Mohamed Seck

**Affiliations:** 1 Faculty of Health Sciences, University Gaston Berger, Saint-Louis, Senegal

**Keywords:** Renal Replacement Threrapy, Renal Dialysis, Africa South of the Sahara

Dear Editor,

We read with interest the article by Smyth A about dialysis indications and the modalities of RRT in elderly patients (1). We would herein underline the particular situation in resource limited areas like sub-Saharan Africa where dialysis access is still very poor and nephrologists have often to make a selection among the growing number of and end-stage renal disease (ESRD) patients who need dialysis (2). We recently reported a high prevalence of chronic kidney disease (CKD) (10.7%) in black African patients aged ≥ 60 years with more than half of them presenting ESRD and having no access to renal replacement therapy (RRT) (3). So discussing indications and dialysis methods may appear as superfluous in a context of few dialysis facilities where elderly patients are in competition with young adults and children who are more likely to take profits from dialysis treatment. In this article, authors recommend to balance short-term RRT survival benefit (hemodialysis or peritoneal dialysis) with possible evitable co-morbidities and unnecessary medicalization (1). We agree with the general rule of proposing dialysis to all ESRD patients regardless of their age but for nephrologists working in countries with poor dialysis access, we think that conservative multidisciplinary management should be encouraged as a socio-culturally and medically efficient alternative for elders with ESRD specially if they have co-morbidities (4, 5). Cohort studies comparing RRT and conservative therapy in elders living resource-limited countries should be performed to precise dialysis indications in these populations.
